# 583. Increasing Diversity, Equity, and Inclusion in the Antimicrobial Stewardship Workforce through the SIDP Certificate Program

**DOI:** 10.1093/ofid/ofae631.178

**Published:** 2025-01-29

**Authors:** Sarah Eudaley, Melissa D Johnson, Meghan N Jeffres, Kenneth Lawrence, Scott J Bergman, Hermsen D Elizabeth, Tracy N Zembles

**Affiliations:** Society of Infectious Diseases Pharmacists, Knoxville, TN; Duke University, Durham, North Carolina; University of Colorado Anschutz Medical Campus, Aurora, CO; Society of Infectious Diseases Pharmacists, Knoxville, TN; Nebraska Medicine, Omaha, Nebraska; Pfizer, Inc., Elkhorn, Nebraska; Children's Wisconsin, Milwaukee, Wisconsin

## Abstract

**Background:**

A diverse workforce provides opportunities for patients to be served by healthcare practitioners sharing a common race, ethnicity, culture, language, or disability. The Society of Infectious Disease Pharmacists (SIDP) aims to increase engagement in an Antimicrobial Stewardship Certificate Program (ASCP) among clinicians identifying with underrepresented minoritized (URM) groups to increase workforce diversity, equity and inclusion; reduce antimicrobial resistance; and improve outcomes.

**Figure:**
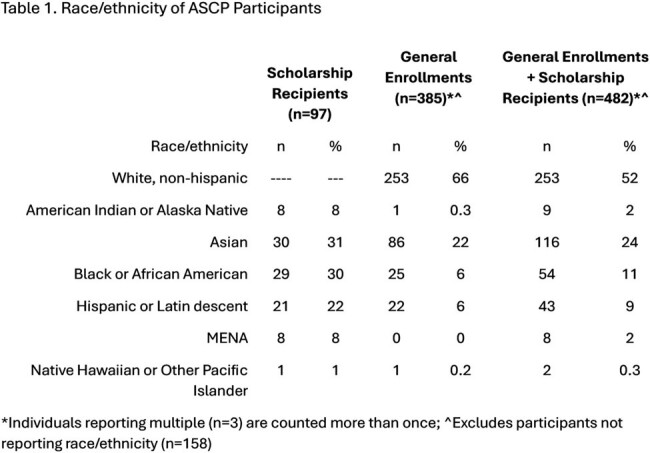

**Methods:**

SIDP updated and redesigned its long-standing ASCP in May 2023. Healthcare professionals identifying as URM were invited to apply for a scholarship to complete the ASCP, with planned enrollment of 130 participants over 3 years. Baseline data collection included demographics, motivation for training, anticipated challenges in completing the ASCP, and confidence in stewardship activities (Likert scale 1-5, with 5 as the highest level). Demographic data prior to May 2023 was not available.

**Figure:**
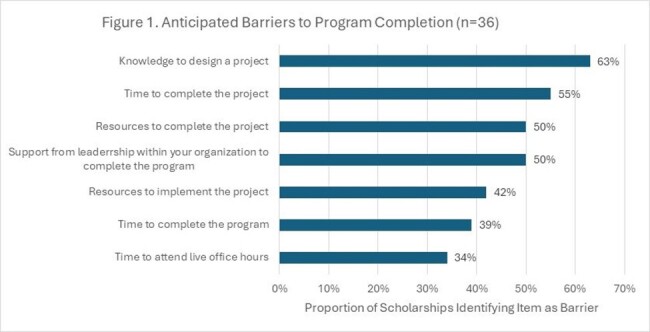

**Results:**

To date, 97 individuals received a scholarship and were enrolled. Most were pharmacists (65%), with allied health professionals comprising the remainder. Participants most commonly identified as Black or African American (29%), Hispanic or Latin descent (21%), and Asian (18%) (Table 1). Considering non-scholarship enrollments (among those reporting race/ethnicity) over the same time-period (n=385), the scholarship increased the proportion of total participants in the program identifying as Black or African American (11% v 6%), Hispanic or Latin descent (9% v 6%), and Middle East or North African (2% v 0%). Among scholarship recipients who started the program and received an entrance survey (n=45), 80% (n=36) responded. The most anticipated challenge for successfully completing the program was knowledge to design a quality improvement project (Figure1). Respondents reported the least confidence in ability to establish an antimicrobial stewardship program (Figure 2).

**Figure:**
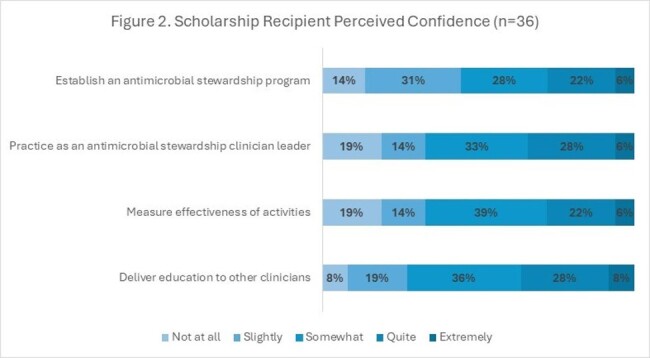

**Conclusion:**

The scholarship program has increased the proportion of URM clinicians in the ASCP, resulting in a potentially more diverse workforce. Identifying and mitigating barriers to completion and assessing participant confidence in stewardship activities will inform additional program development.

**Disclosures:**

**Melissa D. Johnson, PharmD MHS AAHIVP**, Biomeme: Licensed Technology|Scynexis, Inc: Grant/Research Support|UpToDate: Author Royalties **Kenneth Lawrence, BS, PharmD**, Seres Therapeutics: Employee **Hermsen D. Elizabeth, PharmD, MBA**, Pfizer, Inc.: Employee|Pfizer, Inc.: Stocks/Bonds (Public Company)

